# *Toxoplasma gondii* infection possibly reverses host immunosuppression to restrain tumor growth

**DOI:** 10.3389/fcimb.2022.959300

**Published:** 2022-08-31

**Authors:** Jiating Chen, Wenzhong Liao, HongJuan Peng

**Affiliations:** Department of Pathogen Biology, School of Public Health, Guangdong Provincial Key laboratory of Tropical Medicine, Southern Medical University, Guangzhou, China

**Keywords:** *Toxoplasma gondii*, immunotherapy, tumor suppression, avirulent *T. gondii* strains, CD8 + T cell, dendritic cells (DCs)

## Abstract

Tumor cells can successfully escape the host immune attack by inducing the production of immunosuppressive cells and molecules, leading to an ineffective tumor treatment and poor prognosis. Although immunotherapies have improved the survival rate of cancer patients in recent years, more effective drugs and therapies still need to be developed. As an intracellular parasite, *Toxoplasma gondii* can trigger a strong Th1 immune response in host cells, including upregulating the expression of interleukin-12 (IL-12) and interferon-γ (IFN-γ). Non-replicating uracil auxotrophic strains of *T. gondii* were used to safely reverse the immunosuppression manipulated by the tumor microenvironment. In addition to the whole lysate antigens, *T. gondii*-secreted effectors, including *Toxoplasma* profilin, rhoptry proteins (ROPs), and dense granule antigens (GRAs), are involved in arousing the host’s antigen presentation system to suppress tumors. When *T. gondii* infection relieves immunosuppression, tumor-related myeloid cells, including macrophages and dendritic cells (DCs), are transformed into immunostimulatory phenotypes, showing a powerful Th1 immune response mediated by CD8^+^ T cells. Afterwards, they target and kill the tumor cells, and ultimately reduce the size and weight of tumor tissues. This article reviews the latest applications of *T. gondii* in tumor therapy, including the activation of cellular immunity and the related signal pathways, which will help us understand why *T. gondii* infection can restrain tumor growth.

## Introduction

*Toxoplasma gondii* is an obligate intracellular protozoan that infects all mammals and most birds (Jones and Dubey, 2012), and it is estimated that nearly one-third of the world’s population is infected by the parasite (Jones et al., 2001). *T. gondii* strains are conventionally categorized into three types (I, II, and III), which show different virulence traits in mice (Grigg et al., 2001). Due to the vigorous host immune response, *T. gondii* infection is mostly manifested as latent and asymptomatic infection, but it can cause severe toxoplasmosis in immunocompromised patients and birth defects in pregnant women with primary infection (Hoffmann et al., 2012; Torgerson and Mastroiacovo, 2013).

It has been reported more and more recently that microbial infection can enhance the antigen-presentation ability of dendritic cells (DCs), secrete tumor-killing cytokines, and activate innate immunity to act on the tumor microenvironment (TME) (Miller et al., 2002). In 1891, William Coley observed that *Serratia* spp. and *Streptococcus* spp. infection can lead to the regression of sarcomas (Coley, 1991) and since then, more viruses, bacteria, and protozoan pathogens have been found to activate innate immunity to kill tumor cells. These microorganisms include the oncolytic virus *Talimogene laherparepvec* (Andtbacka et al., 2015), yellow fever vaccine 17D (Aznar et al., 2020), cowpea mosaic virus (Lizotte et al., 2016), *Listeria monocytogenes* (Zhou et al., 2018), *Lactobacillus casei* (Ni et al., 2017), *Escherichia coli* (Chowdhury et al., 2019), and *T. gondii.* It has been reported that *T. gondii* infection can suppress the development of many types of tumors, such as Lewis lung carcinoma (Kim et al., 2007), Sarcoma-180 Tumor (Pyo et al., 2010), and fibrosarcoma (Darani et al., 2009). As early as 1977, it was reported that *T. gondii* infection could cause intense inflammatory cell reactions in the brain and around ependymoblastoma in subcutaneously inoculated C57BL/6J (Conley and Remington, 1977). In a study involving 150 patients with breast cancer, prostate cancer, or colon cancer and 120 normal people, patients with low anti-*T. gondii* antibody titers may be associated with a better prognosis, indicating that asymptomatic *T. gondii* infection can stimulate anti-tumor immunity (Seyedeh et al., 2015). In-depth transcriptome-sequencing data show that *T. gondii* infection interferes with gene expression including colorectal cancer, non-small cell lung cancer, and breast cancer pathways, which may ultimately inhibit tumor growth (Lu et al., 2019). Furthermore, in many cancers, *T. gondii* infection is conducive to resisting tumor growth, metastasis, and the development of advanced tumors (Caner, 2021). With the gradual understanding of the anti-tumor effects of *T. gondii* infection, multiple hypotheses have been progressively raised, including hypoxia, avascular necrosis, suppression of tumor angiogenesis, and induction of Th1 immune responses (Hunter et al., 2001). The most fundamental reason may be that the molecular signal pathways regulated by parasites are roughly consistent with those perturbed in the process of carcinogenesis. For instance, *T. gondii* infection can induce natural killer (NK) cells to be converted into innate lymphoid-like cells (ILC1-like cells) in the TME, which means that a group of cells with immune memory function can disseminate throughout the circulation and maintain the production of IFN-γ (Park et al., 2019). However, in the late stage of the acute infection, these hypoxia and avascular necrosis effects gradually vanish when the parasites are finally eliminated, then the remaining tumor cells resumed growth (Hunter et al., 2001). To further exploit *T. gondii’*s immunotherapeutic effects against cancers, we summarized the anti-tumor mechanism of *T. gondii* infection, focusing on the attenuated *T. gondii* strains, the role of *T. gondii* secreted proteins, and the host anti-tumor pathways provoked by *Toxoplasma* infection.

## Avirulent *T. gondii* strains applied in tumor therapy

*T. gondii* disrupt host immunity by secreting virulence factors and successfully parasitizing host cells. As an intracellular parasitic protozoan, *T. gondii* genome encodes a series of enzymes that convert host cell molecules into raw materials for their replication, including two synthetases in the *de novo* biosynthesis pathway of uracil monophosphate nucleotides:carbamoyl phosphate synthetase II (CPS-II) and orotidine-5’-monophosphate decarboxylase (OMPDC) (Fox and Bzik, 2010). Both of them are indispensable for the synthesis of uracil nucleotide (UTP). The non-replicating *Toxoplasma* uracil auxotrophs (NRTUAs) mainly refer to the *T. gondii* mutants with *cps* or *ompdc* knockout. When supplemented with uracil *in vitro*, NRTUAs can normally invade and replicate in the host cells, but they cannot propagate in animals or cells without uracil supply, which is considered to have completely lost virulence. *Cps II* knockout strain (Δ*cps*) can be cleared in mice within approximately 5 days but it still has the ability to significantly inhibit tumor growth, and even regress the established tumors (Dupont et al., 2014). For this reason, NRTUAs are regarded as “immunotherapeutic attenuated vaccine strains”, indicating that non-replicating *Toxoplasma* could be a safe vaccine for cancer therapy (**Table 1**).

**Table 1:** Applications of avirulent *T. gondii* strains in tumor therapy.

Target tumor	Tumor cell strain	Tumor cell inoculation methods	Number of inoculated tumor cells	Mouse species	*Toxoplasma* strain	*Toxoplasma* inoculation methods	Number of inoculated *Toxoplasma*	Time point of *Toxoplasma* therapy	Effective cytokines, immune cells, and signal pathways	References
Ovarian cancer	ID8 cells	i.p.	ID8-Defb29/Vegf-A: 2 × 10^6^ ID8-Vegf-A cells: 1×10^6^	C57BL/6: IL-12p40-/-, IL-12p35-/-, IL-17a-/-, MyD88-/- , OT-1 and Foxp3GFP mice	RH-Δ*cps*	i.p.	2×10^6^	8 and 20 days post tumor cells inoculation (dpi)	CD8^+^ T cell	(Baird et al., 2013b)
Pancreatic cancer	Pan02	i.p.	1 × 10^6^	C57BL/6	RH-Δ*cps*	i.p.	2×10^6^	Survival studies: 5-dose schedule (7, 19, 31, 43, and 55 dpi) and re-challenged 225 days after primary tumor inoculation.	CD4^+^ T cells CD8^+^ T tumor-specific IgG responses	(Sanders et al., 2016)
Melanoma ovarian cancer Lewis Lung carcinoma	B16F10 UpK10 cells Lewis lung tumors	i.d.	B16F10: 1.25×10^5^ UpK10 : 5×10^5^ Lewis lung tumor: 1×10^6^	C57BL/6: IL-12p35-/-, IFN-γ-/- and NOD/SKID/IL2Rγ-/- mice	RH-Δ*cps*	i.t.	1.5×10^7^	9–11 dpi	IL-12, IFN-γ, CXCR3 CD8+ T cell, NK cells Tumor antigen-specific responses	(Baird et al., 2013a)
Breast cancer	4T1	s.c.	10^5^	BALB/c	RH-Δ*ompdc*	i.t.	1-2×10^6^	8 dpi	IL-12 and IFN-γ	(Xu et al., 2021)
Pancreatic cancer	Pan02 cell	i.p.	10^6^	C57BL/6: IL12p35-/-, IFN-γ-/-, MyD88-/-, and CD8a-/- mice	RH-Δ*cps*	i.p.	2×10^6^	Survival studies: 2-dose (7 and 19 dpi), 3-dose (7, 19, and 31 dpi), or 6-dose (7, 8, 11, 12, 24, and 36 dpi) Cytokine analysis: treated with cps at 7 dpi Cellular analysis: treated with cps at 14 dpi	IL12 and IFNγ MyD88 pathway CD8+ T cell	(Sanders et al., 2015)
Pancreatic cancer	Pan02 cell	s.c.	10^6^	C57BL/6	RH-Δ*ompdc*Δ*up*	i.p.	2×10^6^	14, 18, 21, and 28 dpi	IL-12, IFN-γ, CD8+ T cell MyD88 pathway	(Bahwal et al., 2022)
melanoma	B16F10	i.d.	1.25 × 10^5^	C57BL/6	ME49-Δ*ompdc* ME49-Δ*ompdc-*Δ*ldh1* ME49-Δ*ldh1-*Δ*ldh2*	i.t.	105	8, 9, 12, 13, and 16 dpi	IL-12, TNF-α, and IFN-γ	(Li et al., 2021)

i.p. (intraperitoneally), i.d. (intradermally), s.c. (subcutaneously), i.t. (intratumorally).

RH-Δ*cps* preferentially invades CD11c^+^ cells in the microenvironment of ovarian carcinoma, upregulates the levels of the T-cell receptor costimulatory molecules CD80 and CD86, then effectively improves its antigen-presenting efficiency (Baird et al., 2013b). Similarly, RH-Δ*cps* have been reported to invade mature macrophages and DCs, increase the expression of CD80 and CD86, and reprogram the suppressed myeloid cells to alleviate tumor immunosuppression (Sanders et al., 2016). CPS recombinant protein was intratumorally injected to B16F10 murine melanoma to stimulate the anti-tumor immune response mediated by strong CD8^+^ T cells, stimulate systemic anti-tumor immunity, and produce anti-tumor immune memory (Baird et al., 2013a). Furthermore, RH-Δ*cps* can arouse long-term immunity to disseminated pancreatic cancer and prevent the recurrence of highly aggressive tumors to some extent (Sanders et al., 2016). Intraperitoneal injection of the recombinant RH strain with uridine phosphorylase gene and *ompdc* knockout together with anti-PD-1 antibody can markedly reduce the immunosuppressive myeloid-derived suppression in pancreatic tumor-bearing mice (Bahwal et al., 2022). *In situ* inoculation of RH-Δ*ompdc* mutant in 4T1 murine breast tumor can restrict tumor growth and lung metastasis (Xu et al., 2021). NRTUAs therapy can even provide long-lasting protection against the re-challenged pancreatic cancer cells (Sanders et al., 2016).

In addition to NRTUAs, *T. gondii* lactate dehydrogenase (LDH) knockout mutant also presents significant virulence reduction *in vivo* (Xia et al., 2018a). Avirulent ME49-Δ*ldh1* and ME49-Δ*ldh2* can stimulate high level of Th1 immunity and inhibit the growth of melanoma tumors by *in situ* inoculation (Li et al., 2021). The *ldh*-deficient mutant is not only a good vaccine candidate against *T. gondii* infection, but also a valuable biological for tumor treatment (Xia et al., 2018b).

## *T. gondii* molecules participate in anti-tumor immunity in the host

Since the discovery of the potent anti-tumor effect of *T. gondii*, researchers have been committing to analyzing the exact toxoplasmic effectors involved in arousing host anti-tumor immunity. Previous studies reported that *T. gondii* lysate antigen (TLA) showed high tumor-targeting activity in mice and rats (Miyahara et al., 1992b). TLA can also inhibit the growth of the chemically induced (20-methylcholanthrene-induced) tumor cells by intramuscular injection (Miyahara et al., 1992a). TLA treatment also leads to a significant increase in the cytotoxic activity of spleen cells in killing cat FL74 lymphoma cells (Yang et al., 1990). In TLA immunized mice, the expression of CD31 (an angiogenesis marker) is inhibited, and the size and weight of sarcoma-180 tumor decreased (Pyo et al., 2010). Even in athymic nude mice, injection of TLA can inhibit tumor growth through activating myeloid differentiation factor 88 (MyD88) signal in bone marrow macrophages and promoting the expression of interleukin-12 (IL-12) in serum (Pyo et al., 2014). Compared to the DNA components, toxoplasmic proteins show higher stimulating activity in DCs to induce a higher level of IL-12p70 (Mirzaei et al., 2015). These results indicate that TLA may suppress tumor growth through early selective induction of IL-12. Using ammonium sulfate precipitation method and subsequent anion-exchange HPLC to detect the effective TLA components, it is found that one fraction may be responsible for the different proportion of IL-12p70 to IL-10 secreted by the matured DCs, although this fraction could not be further determined (Boghozian et al., 2015). However, it is also pointed out that both live parasites and active invasion are required to induce *T. gondii*-specific CD4^+^ and CD8^+^ T cells responses (Dupont et al., 2014). Certainly, in addition to its anti-tumor effect, TLA also contributes to forming a better immune system to surmount many immune disorders, such as hypersensitivities (Alcantara-Neves et al., 2012).

### *T. gondii* profilin and profilin-like protein

*T. gondii* profilin (*Tg*profilin) is a class of actin-binding protein originally isolated from soluble *T. gondii* antigens (STAg), which plays a powerful role in stimulating immune response and reducing the bacterial, viral, and parasitic burdens (Hedhli et al., 2009). Profilin-like protein (*Tg*PLP) shares significant homology with *Tg*profilin, with a predicted molecular weight of 17.5 kD (Pollard et al., 1994). Some articles do not strictly distinguish between *Tg*profilin and *Tg*PLP; therefore, we temporarily regard *Tg*PLP as a component of *Tg*profilin. *Tg*profilin not only contributes to *T. gondii*’s gliding mobility and invasion to host cells, but also acts as an agonist of Toll-like receptor 11 (TLR11) to increase the production of IL-12 through MyD88 (**Table 2**) (Yarovinsky et al., 2005). *Tg*PLP plays an auxiliary role in the treatment of autologous whole-tumor-cell vaccine by activating the MyD88 pathway, resulting in an increase in the level of antigen-presenting cell markers in bone marrow-derived macrophages, which increases the production of IL-12 and promotes their phagocytosis of tumor cells (Pyo et al., 2016). Significantly, even with the pre-inoculation of profilin or STAg, IFN-γ^-/-^ mice also failed to restrain the growth of the pancreatic tumors, indicating that both IFN-γ and DCs are indispensable for the treatment of pancreatic cancer (Payne et al., 2021).

**Table 2:** Applications of *T. gondii* molecules in tumor therapy in mice.

	*Toxoplasma* strain	*Toxoplasma* protein	Target tumor	Tumor cell strain	Mouse species	Tumor cell inoculation methods	Number of inoculated tumor cells	Effective cytokines, immune cells, and signal pathways	References
*T. gondii* profiling (*Tg*profilin) and profilin-like protein (*Tg*PLP)	N28E2 and RH-88 (Type II and Type I)	STAg and profilin	Pancreatic tumor	From KPC mouse-derived allografted pancreatic tumor model	C57BL/6J	s.c.	–	CD4^+^, CD8^+^, or FOXP3^+^ T cells	(Payne et al., 2021)
					IFN-γ^-/-^ mice and Batf3^-/-^ mice				
*T. gondii* excretory/secretory proteins (ESP)	RH strain (Type I)	ESP	Melanoma	B16F10	C57BL/6	s.c.	2×10^5^	CD4+ CD25+ FOXP3+ T cells (Treg), NK cells	(Jiao et al., 2011)
	RH strain (Type I)	ESP	Lung cancer	Lewis	C57BL/6	s.c.	2×10^5^	CD4+ CD25+ FOXP3+ T cells (Treg)	
*T. gondii* rhoptry proteins (ROPs) and dense granule antigens (GRAs)	RH strain (Type I)	ROP5, ROP17, ROP18, ROP35 or ROP38; GRA2 or GRA12, and GRA24	Ovarian carcinoma	ID8	C57BL/6	i.p.	2 × 10^6^	IL-12, IFN-γ, CD4+, and CD8+ T cells	(Fox et al., 2016)
					IL-12p40^-/-^,				
					IL-12p35^-/-^,				
					MyD88^-/-^, Batf3^-/-^, IFN-γ^-/--^, CD8^-/-^,				
					and MHCII^-/-^				
	PRU strain (Type II)	GRA15	Hepatic carcinoma	Hepa1-6	C57BL/6	s.c.	3 × 10^6^	TNF-α, IL-12, IL-6, IL-10	(Li et al., 2017)
	–	Recombinant GRA8	Colon cancer	HCT116	C57BL/6 and BALB/c	s.c.	1 × 10^6^	–	(Kim et al., 2020)
	RH strain (Type I)	GRA16	Non-small-cell lung carcinoma	H1299	BALB/c	s.c.	2 × 10^6^	NF-κB	(Seo et al., 2020)

i.p. (intraperitoneally), s.c. (subcutaneously).

### *T. gondii* secreted proteins: Excretory/secretory proteins, rhoptry proteins, and dense granule antigens

*T. gondii* releases excretory/secretory proteins (ESP) into the culture medium of cell culture tachyzoites, including components that specifically bind to the serum antibodies of previously infected patients and components with protease activity (Ahn et al., 2001; Saadatnia et al., 2012). Like TLA components, they are the predominant candidate vaccines to activate host immunity and play an anti-tumor role (Rezaei et al., 2019). *T. gondii* ESP can induce apoptosis of human K562 erythroleukemic cells, breast cancer MCF-7 cells, prostate cancer DU145 cells, and other cells, and inhibit the growth of B16F10 melanoma, Lewis lung carcinoma, and prostate cancer (Wu et al., 2012). After subcutaneous inoculation of B16F10 melanoma cells and Lewis lung cancer cells in the right armpit of mice, the proportion of Treg cells (CD4+CD25+Foxp3+) is decreased, and which of NK cells increased significantly in the spleen of mice after inoculation with ESP, and the tumor tissue was significantly smaller than that of the control group (without ESP treatment group) (Jiao et al., 2011; Yu-Meng et al., 2019). The components of ESP are further confirmed by HPLC, including secreted proteins dense granule antigen 2 (GRA2) and GRA5 (Zenner et al., 1999).

For successful invasion, *T. gondii* relies on the proteins sequentially secreted by the secretory organelles, including rhoptry, microneme, and dense granule, namely, rhoptry proteins (ROPs), microneme proteins (MICs), and GRAs, which also function in the host cell signaling and transcription pathways (**Table 2**). Before or after invasion, *T*. *gondii* secretes special proteins to be recruited to the parasitophorous vacuole membrane (PVM) for parasite replication and survival, and then recognized by host cells and to trigger immune responses, including anti-tumor responses (Fox et al., 2016). The deletion of PVM-associated proteins (ROP5, ROP17, ROP18, ROP35, or ROP38) and intra-vacuolar network-associated GRAs (GRA2 or GRA12, and GRA24) significantly weakens the anti-tumor response (Fox et al., 2016). Deleting *rop5* (Δ*rop5*) severely abrogates anti-tumor response in IFN-γ-activated mouse embryonic fibroblasts (Zheng et al., 2013). A recombinant GRA8 peptide can improve mitochondrial transport of GRA8, interact with deacetylae (sirtuin-3), regulate the mitochondria activity, and finally improve the specificity and efficiency of mitochondrial-target therapy for human HCT116 carcinoma (Kim et al., 2020). GRA3 and ROP12 are also PVM-associated proteins, but their depletion does not impair the anti-tumor effect of parasites (Fox et al., 2016). Intratumoral administration of the attenuated RH-Δ*GRA17* strain together with PD-L1 blockade shows a remarkable effect in the treatment of melanoma, Lewis lung carcinoma, and colon adenocarcinoma by upregulating innate and adaptive immune pathways (Zhu et al., 2021). Tumor suppressor gene p53 can be activated by DNA damage and oncogene activation signals, and correspondingly, it is regarded as a dysfunction marker of DNA damage response triggered by oncogenic stress (Bykov et al., 2018). GRA16 is secreted by dense granules and exported to the host cell nucleus through PVM, thereby altering the transcription of host genes (Bougdour et al., 2013). GRA16 interacts with host ubiquitin-specific protease 7 and protein phosphatase 2A to manipulate cell-cycle progression and restore p53 pathway through five putative nuclear sequences (Bougdour et al., 2013). GRA16 also improves the treatment efficacy of irinotecan, a chemotherapeutic drug, by inhibiting the activation of nuclear factor kappa B (NF-κB) in non-small-cell lung carcinoma H1299 cells (Seo et al., 2020). Furthermore, as the main regulator of immune and inflammatory processes, NF-κB is also regarded as a tumor-immunosurveillance factor that mediates the crosstalk of many transcription factors [such as the tumor necrosis factor receptor-associated factors (TRAFs)] and regulates the secretion of cytokines (such as TNFα, IL-1, and IL-6), cell proliferation, and apoptosis (Hoesel and Schmid, 2013). GRA15 can be transported to the cytoplasm of host cells through parasite PVM, interact with TRAFs, activate the NF-κB pathway, and induce IL-12 secretion by murine macrophages (Rosowski et al., 2011; Sangaré et al., 2019). GRA15 also induces IL-1β by continuously activating NF-κB pathway through inflammatory adaptor protein, apoptosis-associated speck-like protein, and caspase-1 (Gov et al., 2013). In addition, the expression of GRA15 in macrophages can induce the differentiation of classically activated macrophage (M1), and then inhibit the growth of hepatocellular carcinoma (Li et al., 2017).

## Host immune signaling pathways involved in *Toxoplasma* triggered anti-tumor responses

**Figure 1:**
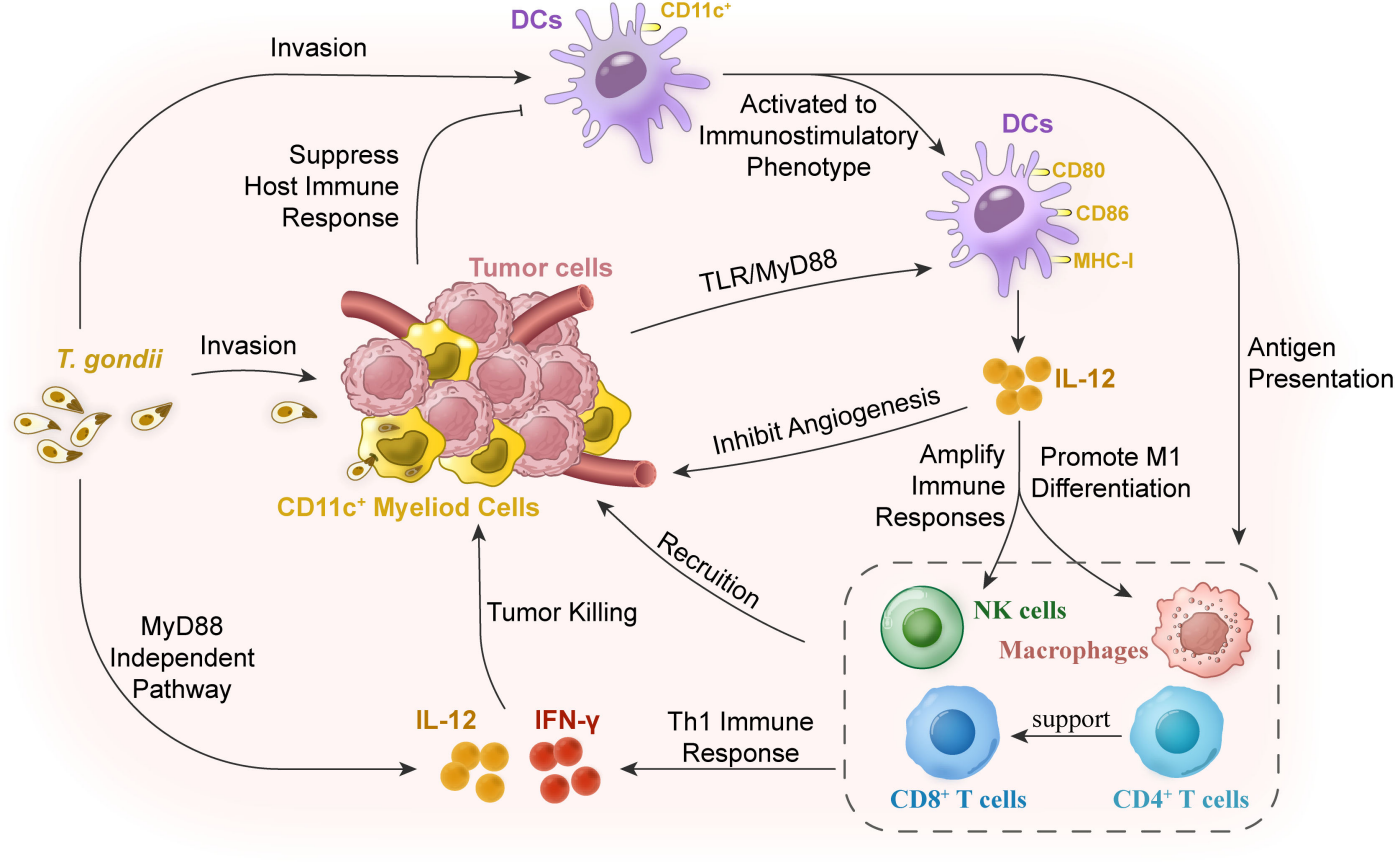
Mechanisms of cellular immunity in reversing immunosuppression for tumor therapy with *T. gondii* vaccination. The myeloid cells in tumor stroma are in an immunosuppressive state, resulting in the decline of antigen-presenting ability of the dendritic cells (DCs) and the subsequent low degree of immune responses. *T. gondii* infection in hosts or *in situ* of the tumor tissues can relieve this low immune response of the myeloid cells in the tumor tissues. DCs in the peripheral blood or tumor tissues can be activated, thus stimulating DC activation and changing the tumor microenvironment by secreting IL-12 to expand the signal of Th1 immune response. *T. gondii* antigens are powerful inducers of IL-12 production, which can trigger the expression of IL-12 through the host’s myeloid differentiation factor 88 (MyD88) signaling pathway. As a cytokine with a wide range of biological activities, IL-12 mainly acts on natural killer (NK) cells, and CD4^+^ and CD8^+^ T lymphocytes. IL-12 is not only a determinant of Th1 cell immune response, but also an angiogenesis inhibitor. Finally, CD8^+^T cells, NK cells, and macrophages were recruited to the tumor tissue to play the role of scavenging tumor cells together with their secreted IL-12 and IFN-γ.

Relieving the suppression of myeloid cells is a major solution to overcome the immunosuppressive disorder of most solid tumors. Significantly, during acute infection, *Toxoplasma* preferentially invades innate myeloid cells, including DCs and monocytes/macrophages, rather than neutrophils or lymphocytes (Channon et al., 2000), and then manipulates the activation of myeloid cells for immune evasion (Baird et al., 2013b). NRTUAs are also observed to preferentially target myeloid cells for invasion (Baird et al., 2013b), and then activate myeloid cells to stimulate strong Th1 and CD8^+^ T-cell responses (Dupont et al., 2014). Up to 25% of DCs and 45% of macrophages in TME were invaded by RH-Δ*cps* when there were rare NRTUA-invaded cells in the spleen and the mesenteric lymph node (Sanders et al., 2015). Meanwhile, *in situ* inoculation of NRTUAs can rapidly recruit CD45^+^ leukocytes (a marker for hematopoietic tumors to detect the differentiation of blood lymphocytes) and CD8^+^ T cells to the tumor (Baird et al., 2013b), and then increase the expression of IFN-γ by CD8^+^ T cells (Sanders et al., 2015) and possibly NK cells (**Figure 1**) (Levy et al., 2011).

### *T. gondii* infection relieves the inhibition of DC maturation

Immature DCs can be recruited to the sites of inflammation or infection, and induced to differentiate into the mature state after encountering microorganisms, and pro-inflammatory or T cell-derived stimuli, accompanied by changes in characteristic phenotypes and functions (Cella et al., 1997). As the center of the immune system, DCs can capture tumor antigens and cross-present them to T cells in tumor-draining lymph nodes, trigger the initial immune response, and present molecules for cancer therapeutic interventions (Palucka and Banchereau, 2012). However, DCs exhibit immature and immunosuppressive phenotypes and even are manipulated to support tumor angiogenesis in the ovarian cancer environment (Curiel et al., 2004). What is worse, DC precursors are recruited into tumors to produce multiple pro-angiogenic factors, then independently assemble neo-vascular systems *in vivo* (Conejo-Garcia et al., 2004). Immature CD11c^+^ DCs can even produce immunosuppressive mediators, such as PD-L1 and phagocytize tumor antigens, leading to the failure of tumor antigens’ cross-presenting to prime T cells (Scarlett et al., 2009). NRTUAs have been applied in the treatment of melanoma and ovarian carcinoma to reverse such tumor-associated immunosuppression (Fox et al., 2013a). Studies have shown that NRTUA invasion can convert tumor-associated CD11c^+^ DCs into an immunostimulatory phenotype, thereby upregulating the expression of IL-12, major histocompatibility antigen I, and T-cell receptor costimulatory molecules CD80 and CD86 (Baird et al., 2013b). These results are consistent with previous reports that show that components of *Toxoplasma* are capable of maturing DCs, which subsequently lead to the activation of CD8^+^ T cells (Motamedi et al., 2009).

### CD8^+^ T-cell activation

The anti-tumor effects in acute inflammation are mainly based on the selective recognition and killing of tumor cells by CD8^+^ T cells (Appay et al., 2008), while it was known that the molecular basis of anti-*Toxoplasma* infection is the strong Th1 immune response and CD8^+^ T-cell immunity (Gazzinelli et al., 1994).

Infection of *T. gondii in vitro* can activate macrophages to kill tumor cells unspecifically, and infection *in vivo* can suppress the tumor growth in immune-deficient mice (Hibbs, 1976). It was also reported that the NRTUA vaccination fails to suppress tumor growth in NOD/SCID/IL-2R γ-chain (NSG) knockout mice due to the lack of B, T, and NK cells in NSG mice (Shultz et al., 2005). This conclusion further illustrates that the anti-tumor effect of NRTUAs cannot be independent of the innate or adaptive immune system. The anti-tumor efficacy of NRTUAs is abrogated with the depletion of CD8^+^ and NK cells, but not CD4^+^ cells (Sanders et al., 2015). It is also reported that CD4^+^ and CD8^+^ T cells are both necessary for the optimal anti-tumor responses (Fox et al., 2016). After NRTUA vaccination, CD8^+^ T cells, CD19^+^ B cells, and CD3^+^ T cells increased rapidly at the inoculation site, followed by a strong Th1 immune response mediated by CD8^+^ T cells (Gigley et al., 2009), which shows great potential in the stimulation of synergistic anti-tumor immune response (Fox et al., 2013b). All these results demonstrate that tumor suppression by NRTUAs requires intact immunity, especially CD8^+^ T cell-mediated immunity. Infection with NRTUAs can not only trigger durable CD8^+^ T cell-mediated immunity, but also trigger humoral immunity of anticancer diseases (Gigley et al., 2009). It has been shown that NRTUA treatment can elicit the expression of circulating pancreatic tumor-specific IgG in Pan02 tumor-bearing mice, and CD4^+^ T cells play a critical role in the re-challenge of Pan02 tumor (Sanders et al., 2016). Taken together, NRTUA treatments can induce a potent Th1-biased immune response and induce long-lasting CD8^+^ T cell-mediated immunity.

### Secretion promotion of IL-12 and IFN-γ

In response to *T. gondii* infection, innate immunity swiftly produces IL-12, which can amplify the response of T cells and NK cells by expressing IFN-γ, promote the differentiation of M1 macrophages, and inhibit angiogenesis, showing a superior anti-tumor response (Gazzinelli et al., 1993; Airoldi et al., 2007; Biswas and Mantovani, 2010). DCs are an essential source of IL-12, and CD8α^+^ DC-deficient mice exhibit severe IL-12 deficiency and high *T. gondii* burden (Dupont et al., 2012). Furthermore, the IL-12 response induced by *Toxoplasma* is mediated by TLR-dependent CD8+ T-cell activation (Pifer and Yarovinsky, 2011). When vaccinated with NRTUAs, IL-12p40 and IL-12p70 increased significantly in the serum of the ovarian cancer-bearing mice, melanoma, and pancreatic cancer (Sanders et al., 2015). RH-Δ*cps* treatment loses its anti-tumor effects in IL-12p40 or IL-12p35 knockout mice (Baird et al., 2013b), indicating that IL-12 production by NRTUA-activated myeloid cells is indispensable for the anti-tumor response. However, direct administration of IL-12 to patients for tumor immunotherapy will cause systemic toxicity (Car et al., 1999). Interestingly, two disparate immunological stages of melanoma treatment have been observed: host IL-12 is not required in the initial stage, but expression by DCs and macrophages is required in the subsequent stage (Baird et al., 2013a). Although the production of MyD88-dependent IL-12 is essential to resist *T. gondii* infection (LaRosa et al., 2008), MyD88 is not necessary for the anti-tumor response of B16F10 melanoma (Baird et al., 2013a) and ovarian cancer (Fox et al., 2016), but is required for pancreatic cancer (Sanders et al., 2015).

If IL-12 alone has limitations in systemic defense against *T. gondii* infection and elimination of malignant tumors, IFN-γ is an indispensable immune stimulation regulator (Pifer and Yarovinsky, 2011), which has been regarded as a strong and trustworthy predictors of treatment success (Burke and Young, 2019). After RH-Δ*cps* treatment, with the increase of tumor-specific CD8^+^ T cells and CD4^+^ T cells in tumors, the IFN-γ level increased 5- to 10-fold in tumor, but there is no significant change in the whole body (Baird et al., 2013a). IFN-γ can still be produced after CD8+ T cells are isolated from theΔ*cps*-treated pancreatic tumor-bearing mice, (Sanders et al., 2015). It was also reported that MyD88 or IL-12 is not required for NRTUA-induced IFN-γ production, because MyD88^-/-^ mice eventually exhibit normal levels of IFN-γ in the absence of IL-12 (Sukhumavasi et al., 2008). Furthermore, IFN-γ^-/-^ mice show defective anti-tumor effects, highlighting the requirement of IFN-γ for anti-tumor response (Fox et al., 2016).

## Conclusion and prospect

Although there has been some progress in understanding the molecular events in tumors and their microenvironment in recent years, as well as in-depth studies on the development of drugs and therapies, the treatment of many cancers is still very passive. Here, we summarize the treatments of non-replicating attenuated *T. gondii* NRTUAs in a murine model of highly aggressive solid tumor, including melanoma, ovarian, and pancreatic. Both RH-Δ*cps* and RH-Δ*ompdc* preferentially invade immunosuppressive CD11c^+^ antigen-presenting cells and restore the ability to efficiently trigger CD8^+^ T-cell responses, showing great potential in the application of tumor immune-chemotherapeutic. The inability of NRTUAs to replicate *in vivo* entrusts them the possibility to be used for anti-tumor therapy. Previous studies also have some interesting conclusions: chronic *T. gondii* infection can delay the development of tumors. In multidrug-resistant tumor cells, infection or incubation with cell lysate of *T. gondii* can promote drug accumulation by reducing ATP-dependent efflux pump activity (Varga et al., 1999). These interesting phenomena also deserve further study. At the same time, their conclusions on the therapeutic effect of non-solid tumors, such as leukemia, are not unified (Caner, 2021). The immune escape mechanism of tumor is complex and precise, but relatively speaking, the signal pathway explored by *T. gondii* in the treatment of tumor is relatively single, because most studies focus on the most critical and classic pathways first, and subsequent studies need to involve more detailed pathways. Among the latest research results, there have been advances in the treatment of colorectal cancer by exosomes from *T. gondii* ME49-infected DCs (ME49-DC-Exo) (Zhu et al., 2022). ME49-DC-Exo can reduce the proportion of M2 macrophages in blood and monocytic myeloid-derived suppressor cells (M-MDSC) in peripheral blood, spleen, and tumor tissue of tumor-bearing mice (Lu et al., 2022). MicroRNA sequencing also identified a series of functional microRNAs, suggesting that the extracting DC exosomes after infection can become a safer means of tumor treatment. Moreover, most studies focus on the cell-mediated immune responses, while the progress of humoral immunity is relatively insufficient. These are the urgent problems to be solved in tumor treatment, especially the comprehensive effect that traditional drugs cannot achieve, which is the focus of in-depth studies on *T. gondii* for tumor therapy in the future.

## Author contributions

All authors contributed to the concept and execution of this review. JC searched the literature, and conceived and wrote the review. WL revised the paper, tables, and graphical abstract. HP critically appraised the literature and made an intellectual contribution to the work. All authors contributed to the article and approved the submitted version.

## Funding

This research was supported by the National Natural Science Foundation of China (81971954 and 81772217), the Science and Technology Planning Project of Guangdong Province (2018A050506038), the Key Project of Guangzhou Science Research (201904020011), and the Basic Research Project of Key Laboratory of Guangzhou (202102100001) to HP.

## Conflict of interest

The authors declare that the research was conducted in the absence of any commercial or financial relationships that could be construed as a potential conflict of interest.

## Publisher’s note

All claims expressed in this article are solely those of the authors and do not necessarily represent those of their affiliated organizations, or those of the publisher, the editors and the reviewers. Any product that may be evaluated in this article, or claim that may be made by its manufacturer, is not guaranteed or endorsed by the publisher.
